# Supplementary material for: Non-destructive monitoring of annual trunk increments by terrestrial structure from motion photogrammetry
Source: PLoS One. 2020 Mar 10;15(3):e0230082. doi: 10.1371/journal.pone.0230082 (PMC7064216; doi:10.1371/journal.pone.0230082)
Supplement: S1 Appendix — (DOCX) [file pone.0230082.s001.docx]

Spruce

data: SpruceReference and SpruceEstiomation

t = 0.082118, df = 29, p-value = 0.9351

alternative hypothesis: true difference in means is not equal to 0

95 percent confidence interval:

-0.3883142 0.4208008

sample estimates:

mean of the differences

0.01624333

--------------------------------------------------------------------------------------------------------------------------------------

Oak

data: OakReference and OakEstiomation

t = -5.7484, df = 29, p-value = 3.183e-06

alternative hypothesis: true difference in means is not equal to 0

95 percent confidence interval:

-1.3553163 -0.6439837

sample estimates:

mean of the differences

-0.99965

--------------------------------------------------------------------------------------------------------------------------------------

Fir

data: FirReference and FirEstiomation

t = 1.9786, df = 29, p-value = 0.05742

alternative hypothesis: true difference in means is not equal to 0

95 percent confidence interval:

-0.01105983 0.66825983

sample estimates:

mean of the differences

0.3286

--------------------------------------------------------------------------------------------------------------------------------------

Beech

data: BeechReference and BeechEstiomation

t = 1.5602, df = 29, p-value = 0.1296

alternative hypothesis: true difference in means is not equal to 0

95 percent confidence interval:

-0.03440185 0.25574851

sample estimates:

mean of the differences

0.1106733

--------------------------------------------------------------------------------------------------------------------------------------
