# Supplementary material for: Non-destructive monitoring of annual trunk increments by terrestrial structure from motion photogrammetry
Source: PLoS One. 2020 Mar 10;15(3):e0230082. doi: 10.1371/journal.pone.0230082 (PMC7064216; doi:10.1371/journal.pone.0230082)
Supplement: Attachment — Submitted filename: Response to Reviewers.docx [file pone.0230082.s002.docx]

Dear John Toland Van Stan II,

Thank you very much for your time and effort. We have answered all questions and comments raised by you and both reviewers.

Editor comment: Both reviewers require substantial revisions before the manuscript can be properly reviewed. After reviewing the manuscript as well, I agree that the methods require (i) greater detail and (ii) improved writing to clarify existing statements. Indeed, there are cases in the methods description where one would need to guess what has been done. On the bright side, both reviewers believe the work has the potential to be of high interest to a broad readership.

Response: We have edited the methods to bring more light to the workflow. Also diagram of whole workflow was added as reviewer 1 suggested. Furthermore we have sent the manuscript to English editing service.

**--------------------------------------------------------------------------------------------------------------------------------------**

Dear Nguyen Van Trung (Reviewer 1),

thank you very much for your time and effort. Your comments were beneficial and important for our manuscript. In following section, we have answered all your comments and edited our manuscript based on them.

Reviewer #1: General comment

The authors conducted a research on monitoring and accuracy assessment of annual trunk increments by terrestrial structure from motion photogrammetry. First, 240 perimeters of four tree species were measured by tape on three height level (0.8 m, 1.3 m and 1.8 m). Then, these perimeters were estimated by terrestrial structure from motion (SfM) photogrammetry techique. All data monitored from after-and before-vegetation season were used to calculate the annual increment. The paired t-test was used to confirm the statically significant difference between annual tree increments calculated from conventional measurements and from terrestrial SfM photogrammetry. Finally, the accuracy assessment of annual trunk increments determined by tape and terrestrial SfM photogrammetry were compared to results measured by increment borer method. Authors remarked “a significant part of forests remains unsuitable if the available terrestrial SfM photogrammetry were to be employed with current accuracy”.

Here I would like offer some comments as follows

Major comments:

Comment 1: Please add a flow chart of experimental steps in Methodology section (consist of image processing, results evaluation and accuracy assessment).

Response 1: The flow chart is now a part of a manuscript. Thank you.

Comment 2: In Table 1, could you add mean of perimeter estimated from terrestrial SfM at each height level of four tree species?

Response 2: The column for overall RMSE for height level was added.

Comment 3: From line 208 to line 217, the paired t-test was used for evaluating both results, please provide more detail of each tree (t=?, df=?, 95% confidence interval=?).

Response 3: The detailed results from t-test are now submitted as S1 appendix and cited in the section.

Comment 4: From line 305 to line 309. Could you show clearly a suitable or unsuitable part if the terrestrial SfM photogrammetry were applied in Fig. 6. Also, please make a legend of blue points and red area in Fig.6.

Response 4: The figure was edited. The colour represent kernel density level. The colour was changed and we have added a line based on RMSE of perimeter estimation. The section was edited accordingly. Thank you

Comment 5: Please add conclusion section to reveal that the results achieved from terrestrial SfM photogrammetry can or NOT be used for monitoring the annual tree increments based on accuracy assessment. And could the terrestrial SfM photogrammetry technique is replaced by the tape measurement?

Response 5: Conclusion is now part of the manuscript. The question raised has been answered. Thank you.

Minor comments:

Comment 6: Line 260. Term “reconstruct of trees” can be replaced by “construct 3D model of trees”.

Response 6: Changed.

Comment 7: Line 267. Term “worst RMSE” can be replaced by “highest RMSE”.

Response 7: Changed.

Comment 8: From line 273 to line 287. Please move to introduction section.

Response 8: Section was moved.

Comment 9: I am not an English native speaker but I think the manuscript should be checked by an English native speaker.

Response 9: We have sent the manuscript to English editing service. Certificate is also submitted.

**--------------------------------------------------------------------------------------------------------------------------------------**

**Reviewer 2**

Dear Reviewer 2,

thank you very much for your comments. In following section, we have answered all your comments and edited our manuscript based on them.

The paper presents an application of structure from motion photogrammetry for measuring trunk diameter increments. This work is novel as it is the first example of measuring trunk increments using a photogrammetric approach and is likely to be of interest to the readers of Plos One.

The paper requires significant improvement in the writing and general presentation before being published. A number of sections are difficult to interpret. For example, the word distract is used instead of subtract in line 110, and units are missing throughout. Nevertheless, the novelty of the work and methods used appear to be sound as such I recommend minor revisions.

Response:

As we have mentioned above the we sent manuscript to English editing. Furthermore the workflow of whole methodology was added to make it more clear.

Other comments,

Comment 1: Some of the methods are not fully described, for example,

- line 146 "To calculate initial diameter and position of the tree we used the circle fitting algorithm [15]." The circle fitting method is not outlined and reference 15 is a comparison of multiple methods.

Response 1: Clarification has been added. The section was rewritten to bring more light to the workflow.

Comment 2: - line 153, how was the diameter derived from the polygon?

Response 2: We have recalculated the diameter from perimeter. The clarification was added within the section. Thank you.

Comment 3: Figure 2 should provide a scale bar for each point cloud

Response 3: Scale for each point cloud for all three axis was added.

Comment 4: The results often repeat the methods

Response 4: The results section was edited in a way to decrease the repetition.
